# Supplementary material for: Forest fire threatens global carbon sinks and population centres under rising atmospheric water demand
Source: Nat Commun. 2022 Nov 22;13:7161. doi: 10.1038/s41467-022-34966-3 (PMC9684135; doi:10.1038/s41467-022-34966-3)
Supplement: Supplementary file 1 — Supplementary Information [file 41467_2022_34966_MOESM1_ESM.pdf]

Supplementary Information – Forest fire threatens global carbon sinks and population centres under rising atmospheric water demand

Hamish Clarke, Rachael H. Nolan, Victor Resco De Dios, Ross Bradstock, Anne Griebel, Shiva Khanal, Matthias M. Boer

Supplementary Note 1 | Generalised linear model performance and flammability thresholds

Supplementary Fig. 1 | Accuracy of generalised linear models for the probability of fire as a function of VPD

Supplementary Table 1 | Generalised linear model results

Supplementary Fig. 2 | VPD thresholds (kPa) above which the probability of fire exceeds 50%

Supplementary Table 2 | Generalised linear model results for forest biome groups

Supplementary Fig. 3 | The mean annual frequency of VPD threshold exceedances (days)

Supplementary Fig. 4 | Projected change in the mean annual frequency of days exceeding the VPD thresholds by 2026-2045 under RCP8.5

Supplementary Fig. 5 | Projected change in the mean annual frequency of days exceeding the VPD thresholds by 2026-2045 under RCP4.5

Supplementary Fig. 6 | Projected change in the mean annual frequency of days exceeding the VPD thresholds by 2081-2100 under RCP4.5

Supplementary Table 3 | Selected study area properties

Supplementary Table 4 | Regions covered by sub-continental windows

Supplementary Table 5 | Global climate models used in this study

Supplementary Fig. 7 | Range of future climate change in selected climate models

Supplementary Fig. 8 | Similarity between presence and absence points

Supplementary Fig. 9 | The correlation between monthly area burnt and monthly frequency of VPD threshold exceedances (days)

Supplementary Fig. 10 | Projected relative change in the mean annual frequency of days exceeding the VPD thresholds by 2026-2045 under RCP8.5

Supplementary Fig. 11 | Projected relative change in the mean annual frequency of days exceeding the VPD thresholds by 2081-2100 under RCP8.5

Supplementary Fig. 12 | Projected relative change in the mean annual frequency of days exceeding the VPD thresholds by 2026-2045 under RCP8.5

Supplementary Fig. 13 | Projected relative change in the mean annual frequency of days exceeding the VPD thresholds by 2081-2100 under RCP8.5

Supplementary Note 1 | Generalised linear model performance and flammability thresholds

Daily VPD values were generally greater (i.e. distribution shifted to right) on fire days compared to non-fire days for almost all combinations of forest biome and sub-continental window, with most models predicting fire on the basis of VPD performing better than chance (Supplementary Fig. 1; Supplementary Table 1). The best performing models (i.e. accuracy > 0.8) represent 27% of biome-window combinations (19 of 70), 40% of forest area (21.6m of 54.0m km^2) and 45% of mean annual area burnt (0.34m of 0.76m km^2). The worst performing models (accuracy < 0.6) represent 19% of biome-window combinations (13 of 70), 14% of forest area (7.8m of 54.0m km^2) and 6% of mean annual area burnt (0.05m of 0.76m km^2).

The VPD threshold was highest in subtropical and tropical biomes (median 2.65 kPa), followed by the Mediterranean biomes (median 2.31 kPa), with temperate and boreal biomes (median 1.26 kPa) having the lowest values. For individual biomes, the highest VPD thresholds were found in tropical and subtropical dry broadleaf forests (median 3.05 kPa), while the lowest were in boreal forests and taiga (median 1.11 kPa).

There was a negative relationship between model accuracy and the annual frequency of days exceeding the VPD threshold ($R^2 = 0.65$) with the worst performing models (accuracy < 0.6) all at the upper end of the distribution of days over threshold per year (> 115 days). Model accuracy had little to no relationship with VPD threshold value ($R^2 = 0.14$), biome area ($R^2 = 0.04$), mean annual area burnt ($R^2 = 0.08$) or mean annual fraction of biome burnt ($R^2 = 0.00$).

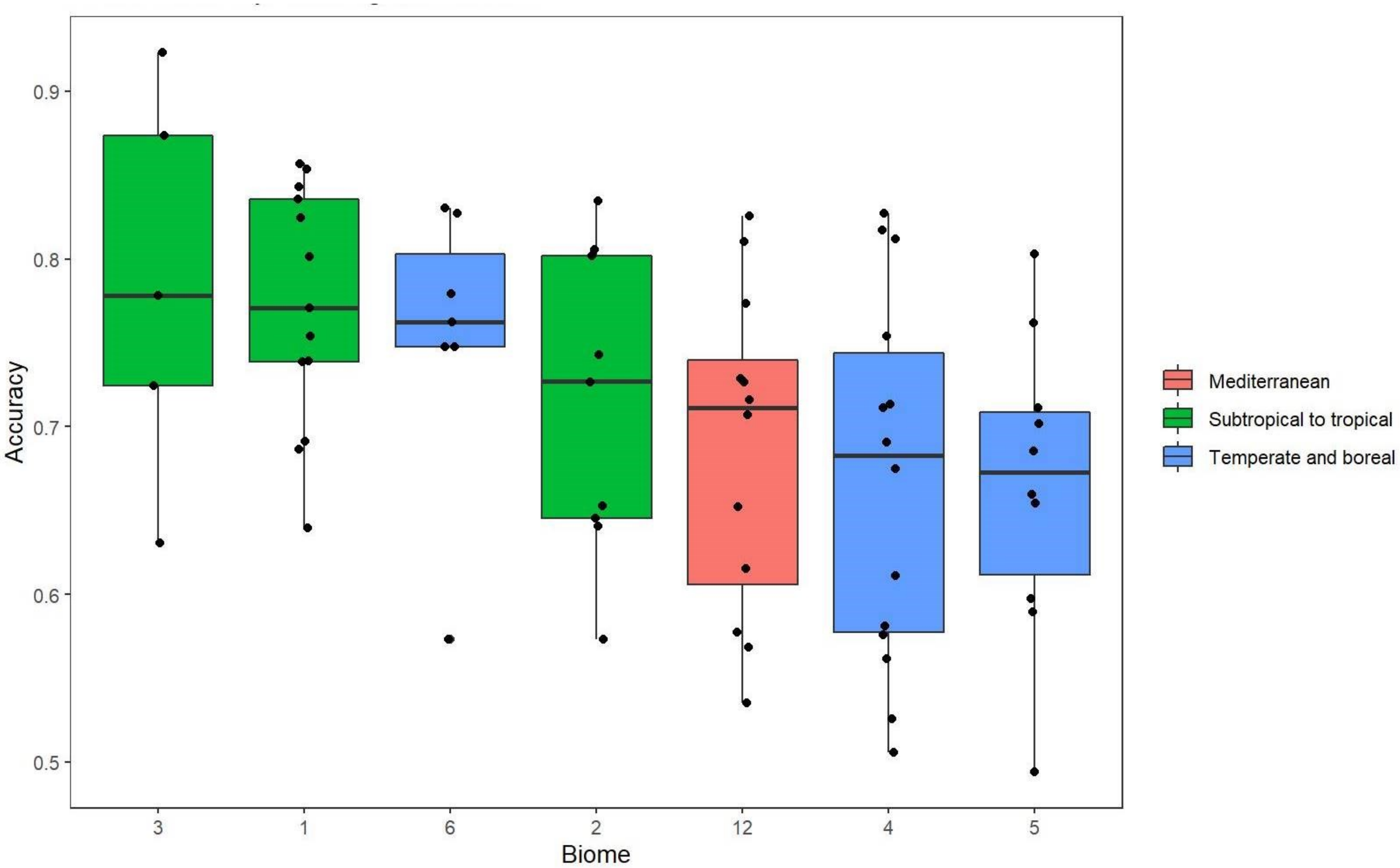

Supplementary Fig. 1 | Accuracy of generalised linear models for the probability of fire as a function of VPD. Each dot represents a combination of forest biome and sub-continental window, boxplots represent forest biomes and are shown in descending order by median VPD threshold (center line, median; box limits, upper and lower quartiles; whiskers, 1.5x interquartile range; points, outliers). Forest biomes are further classified into three biome groups. Biomes are: Tropical and Subtropical Moist Broadleaf Forests (1), Tropical and Subtropical Dry Broadleaf Forests (2), Tropical and Subtropical Coniferous Forests (3), Mediterranean Forests, Woodlands, and Scrub (12), Temperate Broadleaf and Mixed Forests (4), Temperate Coniferous Forests (5), Boreal Forests/Taiga (6).

Supplementary Table 1 | Generalised linear model results. Each forest biome was represented in at least five sub-continental windows. For sample size (i.e. the number of burnt pixels), the VPD at which the probability of fire is 50% ($VPD_{P=50}$), days per year over $VPD_{P=50}$, model accuracy and deviance explained, the median, minimum and maximum across all sub-continental windows within that biome are shown. See Supplementary Table 4 for sub-continental window legend.

Biome group	Biome	Sub-continental window	Sample size (all years)	$VPD_{P=50}$ (kPa)	Annual days > $VPD_{P=50}$	Model accuracy	Deviance explained (%)
Subtropical to tropical	Tropical and Subtropical Moist Broadleaf Forests	3, 4, 5, 6, 9, 10, 11, 12, 13, 14, 18, 19, 20	980,702 (1,754 – 3,925,341)	2.46 (1.58 – 3.49)	52 (0 – 121)	0.77 (0.64 – 0.86)	30 (4 – 47)
Subtropical to tropical	Tropical and Subtropical Dry Broadleaf Forests	3, 4, 5, 6, 12, 13, 14, 18, 19	289,556 (13,332 – 1,995,135)	3.05 (2.70 – 4.04)	80 (1 – 139)	0.73 (0.57 – 0.83)	20 (2 – 37)
Subtropical to tropical	Tropical and Subtropical Coniferous Forests	3, 4, 15, 18, 19	64,499 (3,169 – 547,779)	2.54 (1.46 – 3.65)	64 (23 – 92)	0.78 (0.63 – 0.92)	26 (7 – 68)
Mediterranean	Mediterranean Forests, Woodlands, and Scrub	2, 3, 4, 6, 7, 8, 9, 10, 11, 13, 15, 20	16,670 (256 – 218,851)	2.31 (1.44 – 4.35)	107 (66 – 158)	0.71 (0.54 – 0.83)	17 (0 – 46)
Temperate and boreal	Temperate Broadleaf and Mixed Forests	2, 3, 4, 6, 7, 8, 9, 11, 15, 16, 18, 19, 20, 21	26,802 (210 – 730,408)	1.30 (0.91 – 2.55)	123 (12 – 197)	0.68 (0.51 – 0.83)	15 (1 – 40)
Temperate and boreal	Temperate Coniferous Forests	2, 3, 4, 8, 9, 10, 15, 16, 18, 19	11,610 (1,379 – 542,221)	1.54 (0.90 – 2.04)	89 (0 – 181)	0.67 (0.49 – 0.80)	16 (0 – 34)
Temperate and boreal	Boreal Forests/Taiga	1, 2, 3, 8, 15, 16, 17	369,707 (15,194 – 3,887,837)	1.11 (0.72 – 1.37)	73 (44 – 115)	0.76 (0.57 – 0.83)	31 (4 – 45)

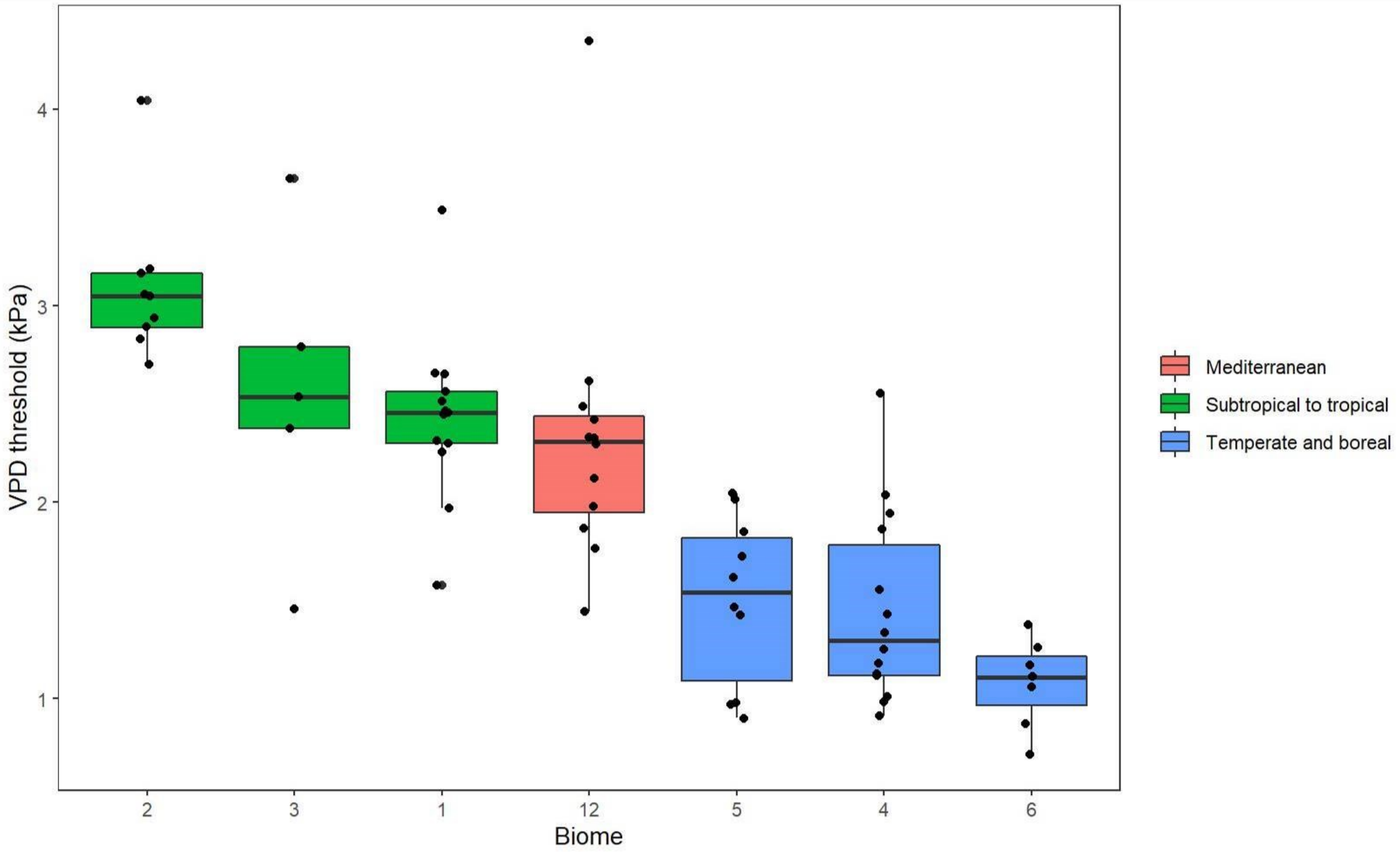

Supplementary Fig. 2 | VPD thresholds (kPa) above which the probability of fire exceeds 50%. Values are derived from generalised linear modelling. Each dot represents a combination of forest biome and sub-continental window, boxplots represent forest biomes and are shown in descending order by median VPD threshold (center line, median; box limits, upper and lower quartiles; whiskers, 1.5x interquartile range; points, outliers). Forest biomes are further classified into three biome groups. Biomes are: Tropical and Subtropical Moist Broadleaf Forests (1), Tropical and Subtropical Dry Broadleaf Forests (2), Tropical and Subtropical Coniferous Forests (3), Mediterranean Forests, Woodlands, and Scrub (12), Temperate Broadleaf and Mixed Forests (4), Temperate Coniferous Forests (5), Boreal Forests/Taiga (6).

Supplementary Table 2 | Generalised linear model results for forest biome groups. Table shows median (minimum – maximum) for VPD at which the probability of fire is 50% ($VPD_{P=50}$), the annual frequency of days exceeding $VPD_{P=50}$. See Supplementary Table 3 for mapping of biomes to biome groups.

Biome group	$VPD_{P=50}$ (kPa)	Annual days > $VPD_{P=50}$
Subtropical & tropical	2.65 (1.46 – 4.04)	61 (0 – 139)
Mediterranean	2.31 (1.44 – 4.35)	107 (66 – 158)
Temperate and boreal	1.26 (0.72 – 2.55)	86 (0 – 197)

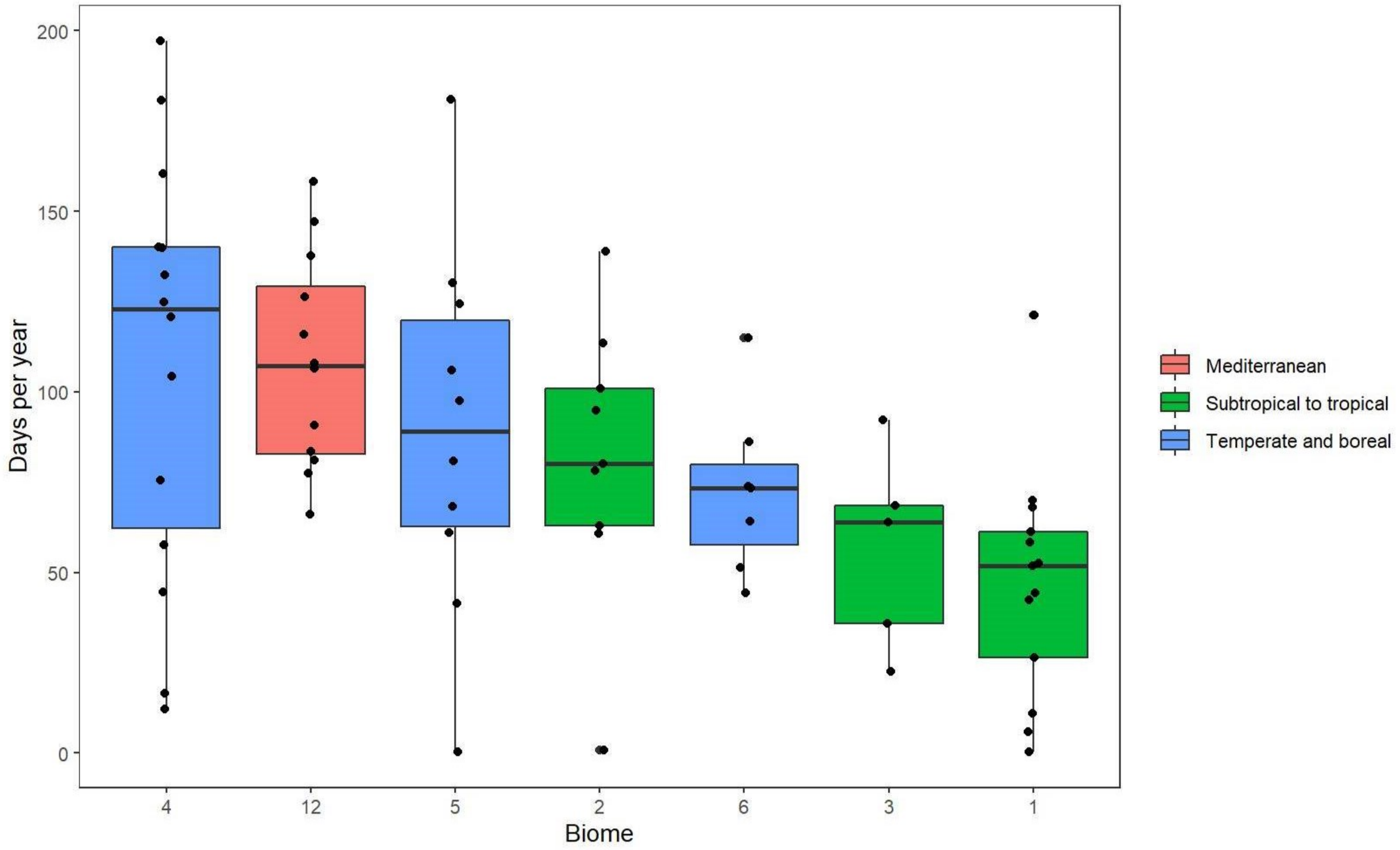

Supplementary Fig. 3 | The mean annual frequency of VPD threshold exceedances (days). Each dot represents a combination of forest biome and sub-continental window, boxplots represent forest biomes and are shown in descending order by median days over VPD threshold (center line, median; box limits, upper and lower quartiles; whiskers, 1.5x interquartile range; points, outliers). Forest biomes are further classified into three biome groups. Biomes are: Tropical and Subtropical Moist Broadleaf Forests (1), Tropical and Subtropical Dry Broadleaf Forests (2), Tropical and Subtropical Coniferous Forests (3), Mediterranean Forests, Woodlands, and Scrub (12), Temperate Broadleaf and Mixed Forests (4), Temperate Coniferous Forests (5), Boreal Forests/Taiga (6).

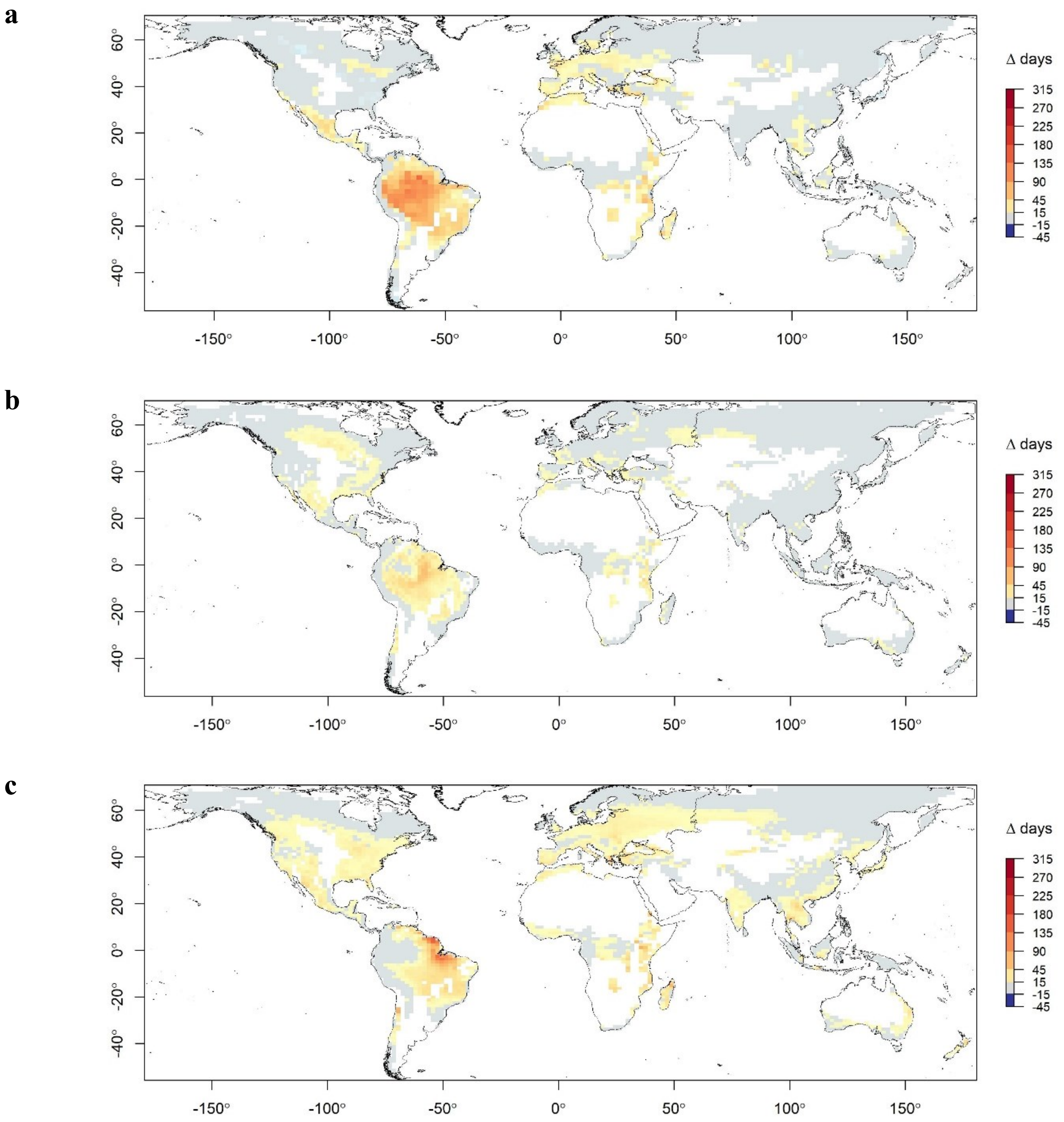

Supplementary Fig. 4 | Projected change in the mean annual frequency of days exceeding the VPD thresholds by 2026-2045 under RCP8.5 for the GFDL-CM3 (a), CNRM-CM5 (b) and ACCESS1.0 (c) models. The white areas indicate non-forest land.

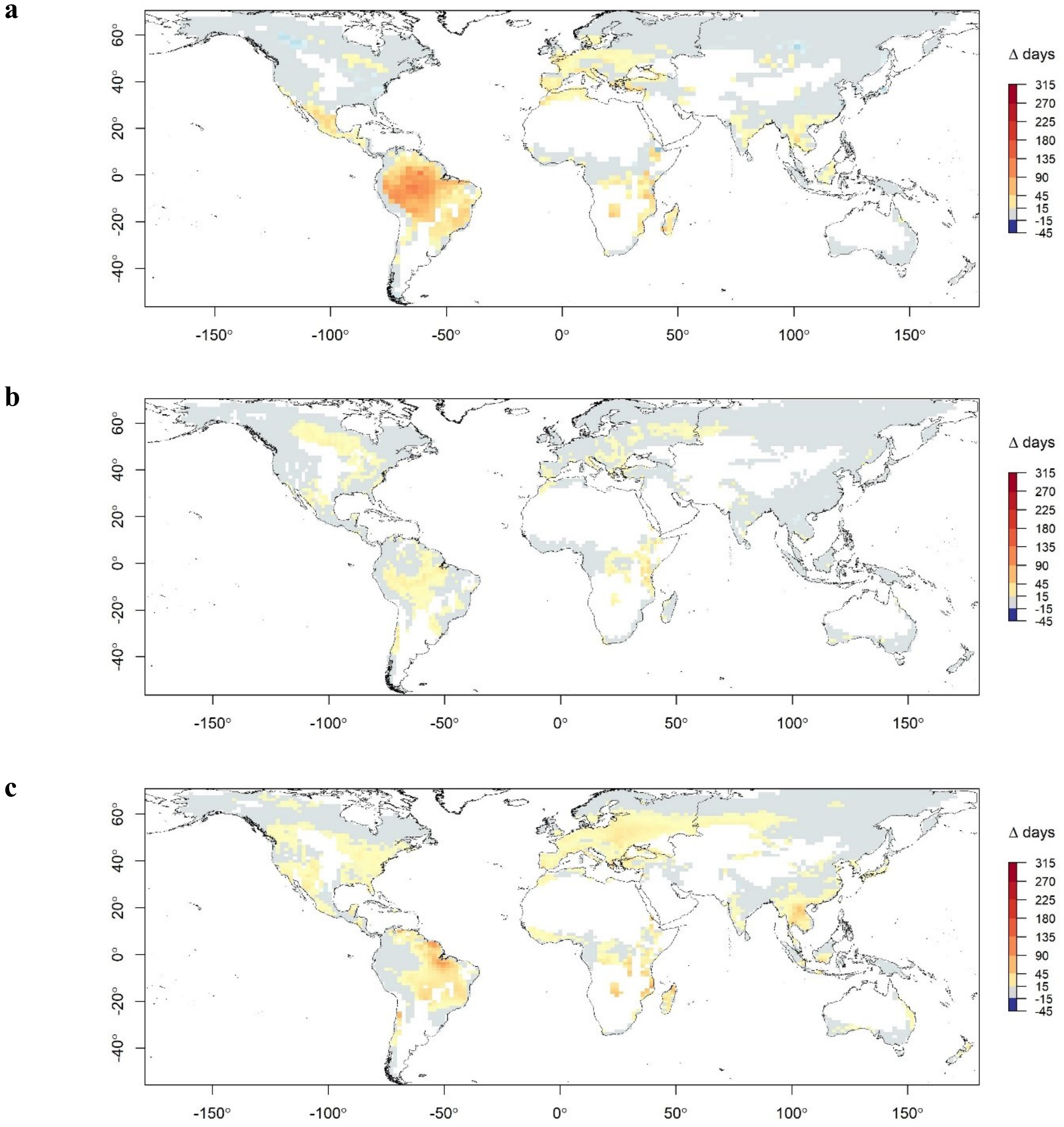

Supplementary Fig. 5 | Projected change in the mean annual frequency of days exceeding the VPD thresholds by 2026-2045 under RCP4.5 for the GFDL-CM3 (a), CNRM-CM5 (b) and ACCESS1.0 (c) models. The white areas indicate non-forest land.

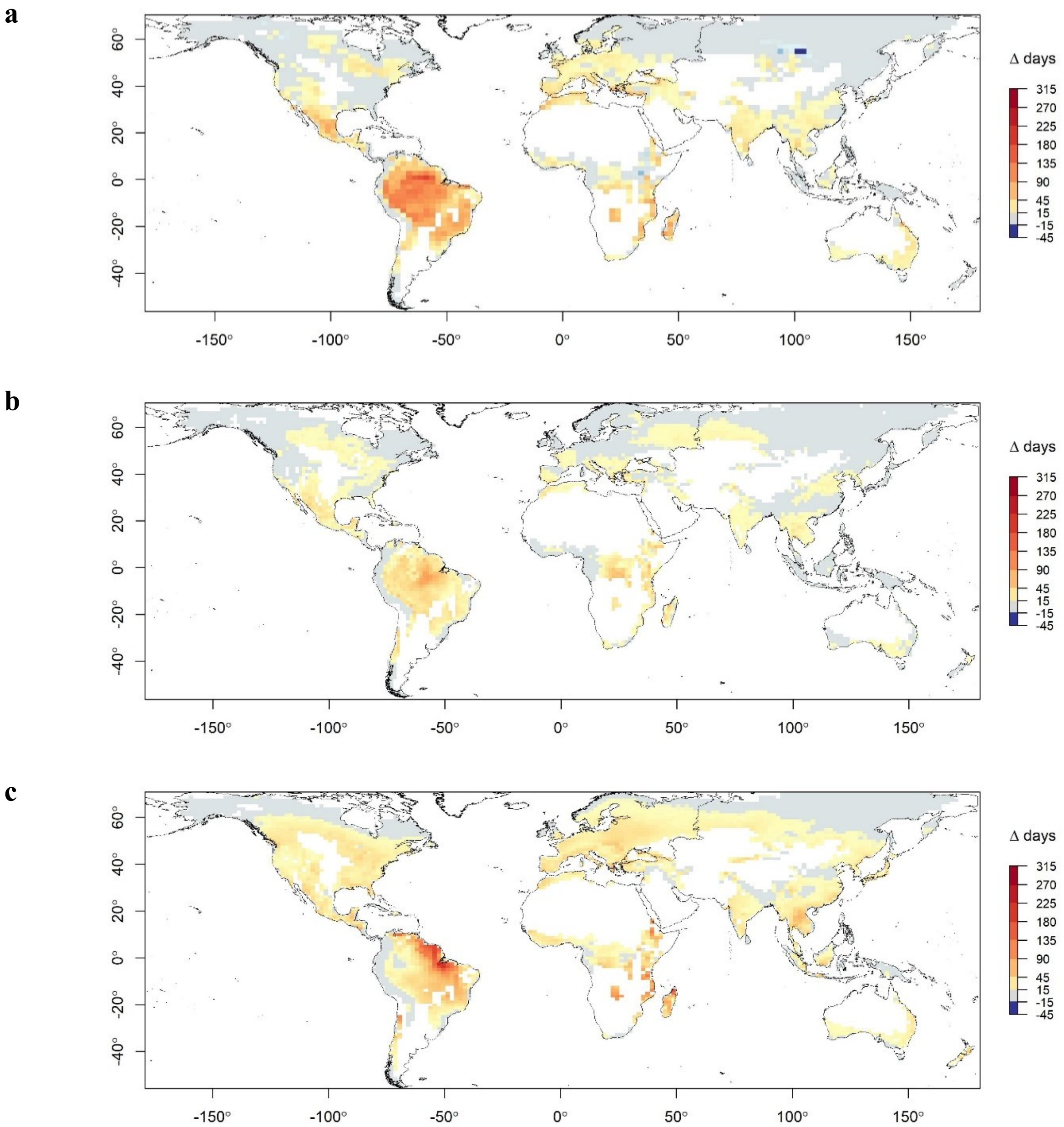

Supplementary Fig. 6 | Projected change in the mean annual frequency of days exceeding the VPD thresholds by 2081-2100 under RCP4.5 for the GFDL-CM3 (a), CNRM-CM5 (b) and ACCESS1.0 (c) models. The white areas indicate non-forest land.

Supplementary Table 3 | Selected study area properties. Each forest biome was represented in at least five sub-continental windows. For area, burnt area and percentage burnt, the median, minimum and maximum across all sub-continental windows within that biome are shown. See Supplementary Table 4 for sub-continental window legend.

Biome group	Biome (number)	Sub-continental window	Area (km^2)	Mean annual area burnt (km^2)	Mean annual % burnt
Subtropical and tropical	Tropical and Subtropical Moist Broadleaf Forests (1)	3, 4, 5, 6, 9, 10, 11, 12, 13, 14, 18, 19, 20	605,071 (15,912 – 6,164,016)	24,493 (44 – 98,034)	2.98 (0.28 – 20.04)
Subtropical and tropical	Tropical and Subtropical Dry Broadleaf Forests (2)	3, 4, 5, 6, 12, 13, 14, 18, 19	204,108 (5,813 – 544,657)	7,232 (333 – 49,828)	3.55 (0.37 – 29.64)
Subtropical and tropical	Tropical and Subtropical Coniferous Forests (3)	3, 4, 15, 18, 19	73,495 (15,610 – 531,906)	1,611 (79 – 13,681)	1.79 (0.51 – 2.57)
Mediterranean	Mediterranean Forests, Woodlands, and Scrub (12)	2, 3, 4, 6, 7, 8, 9, 10, 11, 13, 15, 20	44,780 (2,631 – 927,579)	416 (6 – 5,466)	0.61 (0.18 – 3.79)
Temperate and boreal	Temperate Broadleaf and Mixed Forests (4)	2, 3, 4, 6, 7, 8, 9, 11, 15, 16, 18, 19, 20, 21	415,773 (544 – 2,435,773)	669 (5 – 18,242)	0.26 (0.00 – 3.04)
Temperate and boreal	Temperate Coniferous Forests (5)	2, 3, 4, 8, 9, 10, 15, 16, 18, 19	307,215 (7,645 – 1,439,572)	290 (34 – 13,542)	0.72 (0.07 – 1.89)
Temperate and boreal	Boreal Forests/Taiga (6)	1, 2, 3, 8, 15, 16, 17	934,722 (326,799 – 4,831,481)	9,233 (379 – 97,097)	0.81 (0.05 – 2.01)

Supplementary Table 4 | Regions covered by sub-continental windows.

Window	Region
1	Alaska
2	Canada
3	USA (Conterminous)
4	Central America
5	South America (North)
6	South America (Central)
7	South America (South)
8	Europe
9	West and North Africa
10	Central and North Africa
11	East Africa and Arabian Peninsula
12	Southern Africa (North)
13	Southern Africa (South)
14	Madagascar
15	Russia and Central Asia 1
16	Russia and Central Asia 2
17	Russia (Kamchatka)
18	South Asia
19	South East Asia
20	Australia
21	New Zealand

Supplementary Table 5 | Global climate models used in this study.

	Atmospheric grid resolution		Institution	Reference
Model	Latitude	Longitude		
ACCESS1.0	1.25	1.875	CSIRO (Commonwealth Scientific and Industrial Research Organisation, Australia), and BOM (Bureau of Meteorology, Australia)	Ackerley and Dommenget (2016)
CNRM-CM5	1.4008	1.4008	Centre National de Recherches Météorologiques / Centre Européen de Recherche et Formation Avancées en Calcul Scientifique	Voldoire et al. (2013)
GFDL-CM3	2	2.5	Geophysical Fluid Dynamics Laboratory	Griffies et al. (2011)

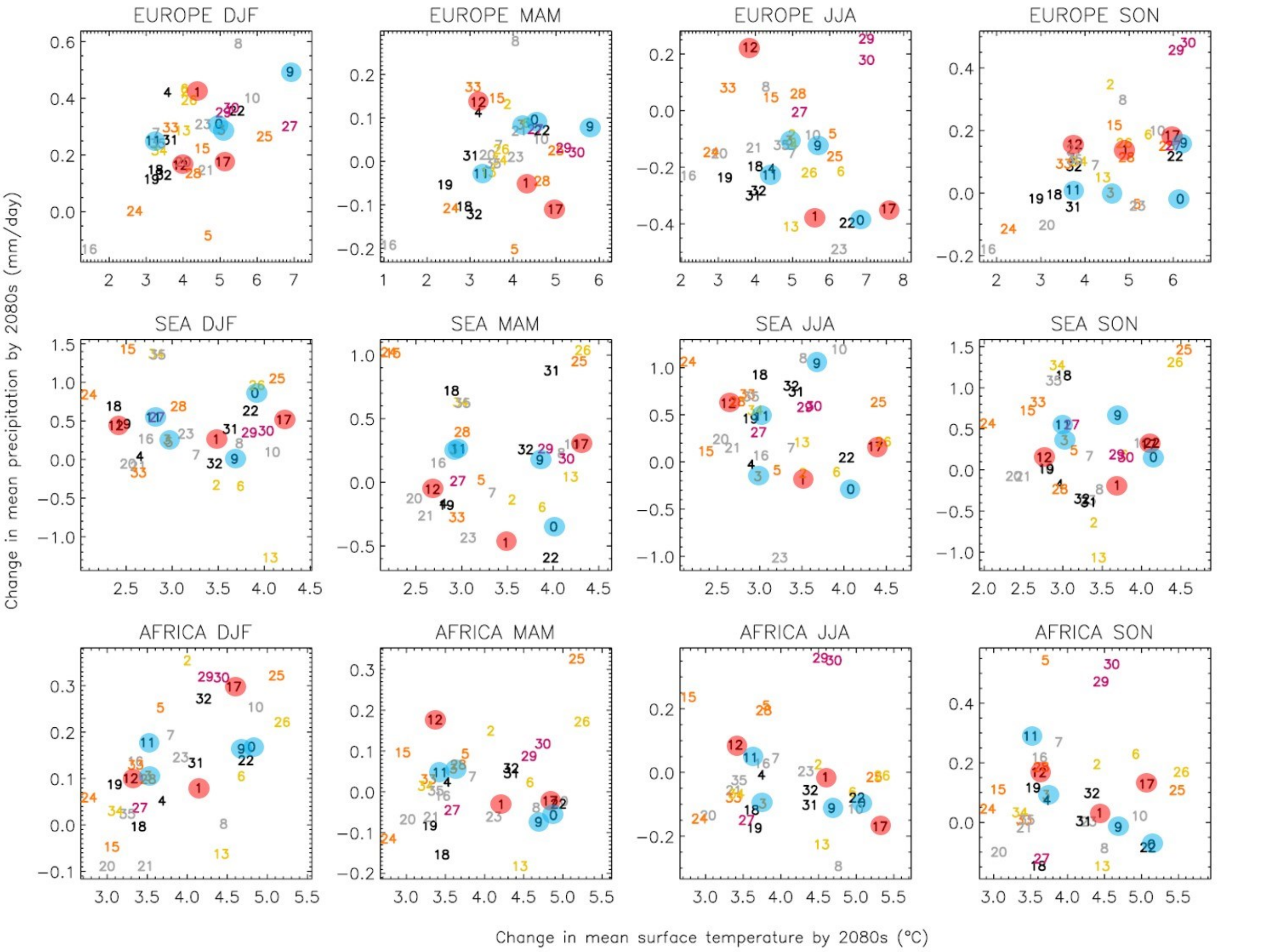

Supplementary Fig. 7 | Range of future climate change in selected climate models. Markers show seasonal mean temperature and precipitation in Europe, South East Asia (SEA) and Africa from the full CMIP5 model ensemble (adapted from Fig 11 in McSweeney et al. 2015). We selected three models based on skill, independence and the ability to span the range of future changes in climate. We have added coloured circles to the plot showing the three models used in this study (red) and four additional models that also met selection criteria but for which model output was not available (blue). Considering actual (red) and potential (blue) models, the actual models span the full range of precipitation changes in 5 out of 12 cases, the full range of temperature changes in 7 out of 12 cases, at least 75% of the range of precipitation changes in 9 out of 12 cases and at least 75% of the range of temperature changes in 11 out of 12 cases.

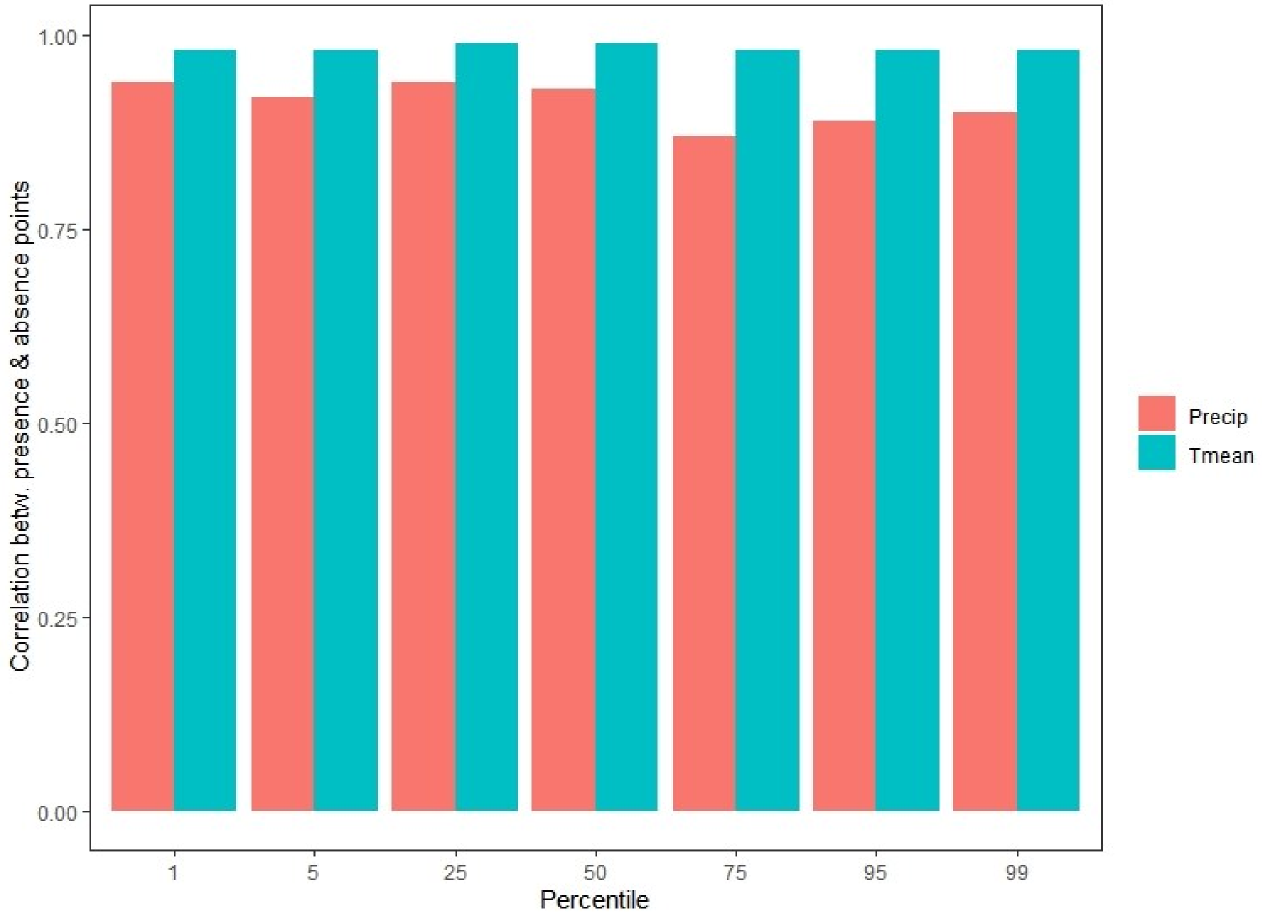

Supplementary Fig. 8 | Similarity between presence and absence points. Bars show correlation between climate (mean annual total precipitation, mean annual temperature) of presence and absence points across all combinations of forest biome and sub-continental window (n=70). Climate data is from WorldClim v2.1 (1970-2000) at 10 minute (~18 km) spatial resolution (Fick & Hijmans 2017).

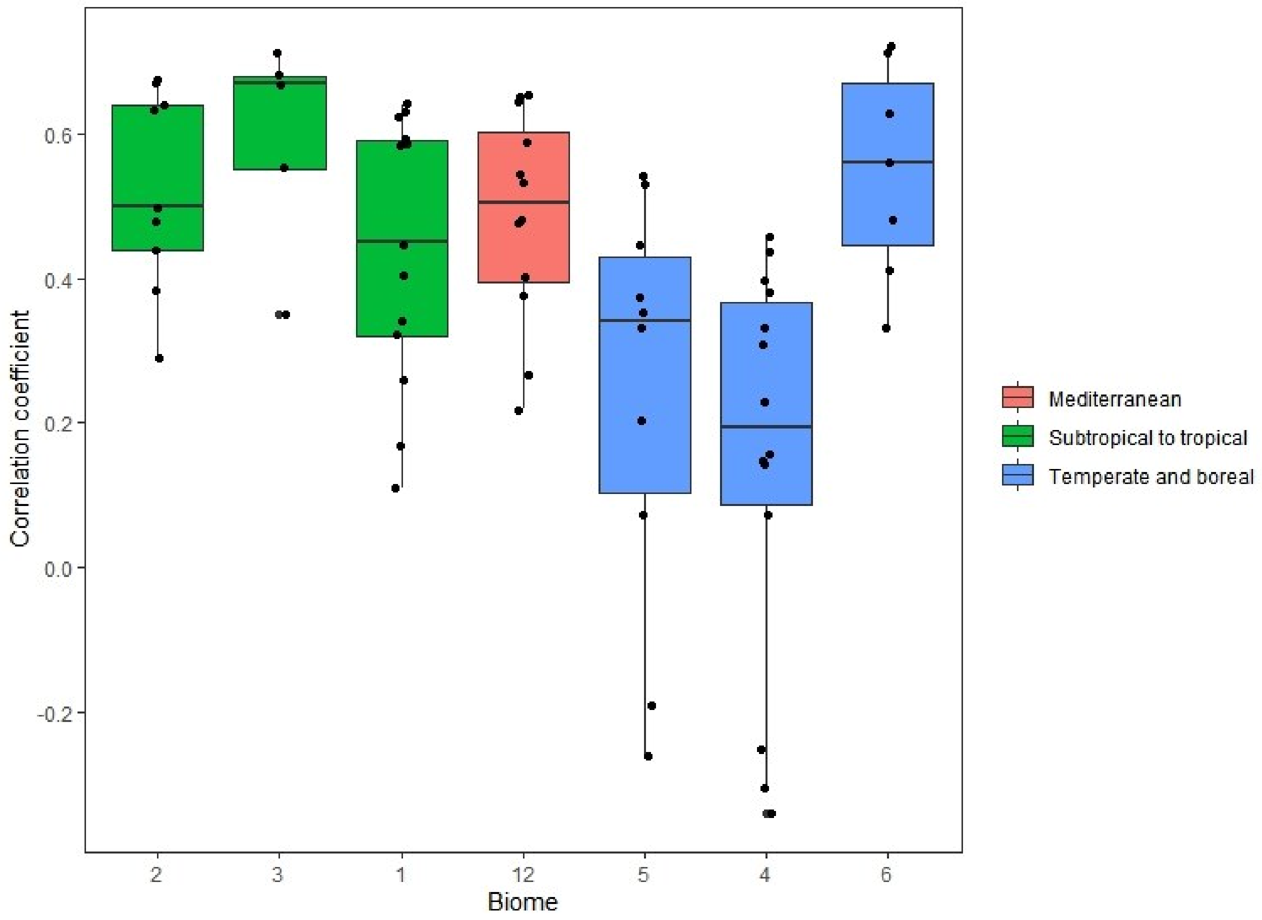

Supplementary Fig. 9 | The correlation between monthly area burnt and monthly frequency of VPD threshold exceedances (days). Each dot represents a combination of forest biome and sub-continental window, boxplots represent forest biomes and are shown in descending order by median days over VPD threshold (center line, median; box limits, upper and lower quartiles; whiskers, 1.5x interquartile range; points, outliers). Forest biomes are further classified into three biome groups. Biomes are: Tropical and Subtropical Moist Broadleaf Forests (1), Tropical and Subtropical Dry Broadleaf Forests (2), Tropical and Subtropical Coniferous Forests (3), Mediterranean Forests, Woodlands, and Scrub (12), Temperate Broadleaf and Mixed Forests (4), Temperate Coniferous Forests (5), Boreal Forests/Taiga (6).

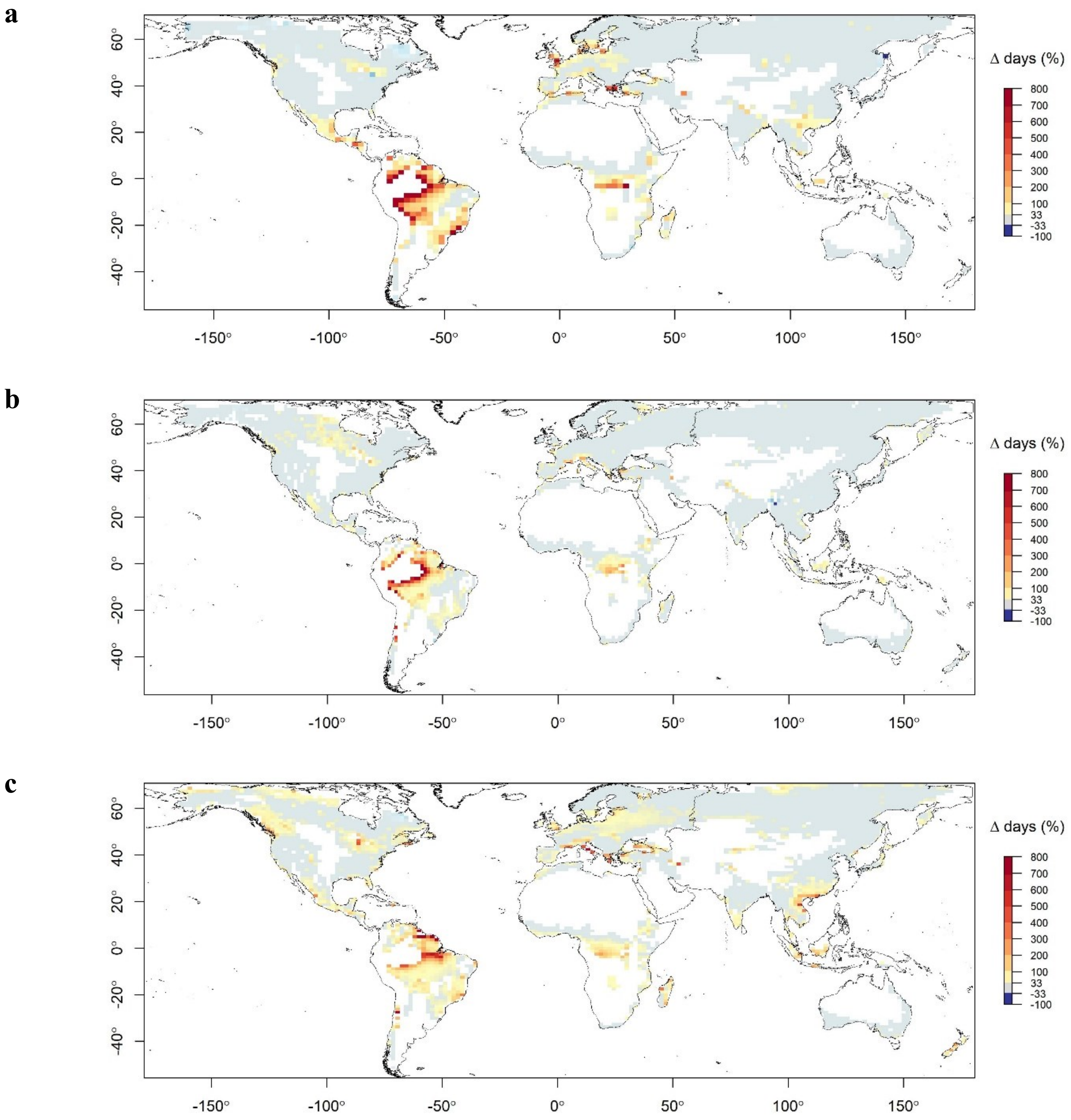

Supplementary Fig. 10 | Projected relative change in the mean annual frequency of days exceeding the VPD thresholds by 2026-2045 under RCP8.5 for the GFDL-CM3 (a), CNRM-CM5 (b) and ACCESS1.0 (c) models. To resolve the majority of the distribution of relative change values, outliers are minimised by capping the scale at 800% (the highest value is 4600%) and omitting pixels with fewer than 5 days per year currently exceeding VPD thresholds. The white areas indicate non-forest land.

a

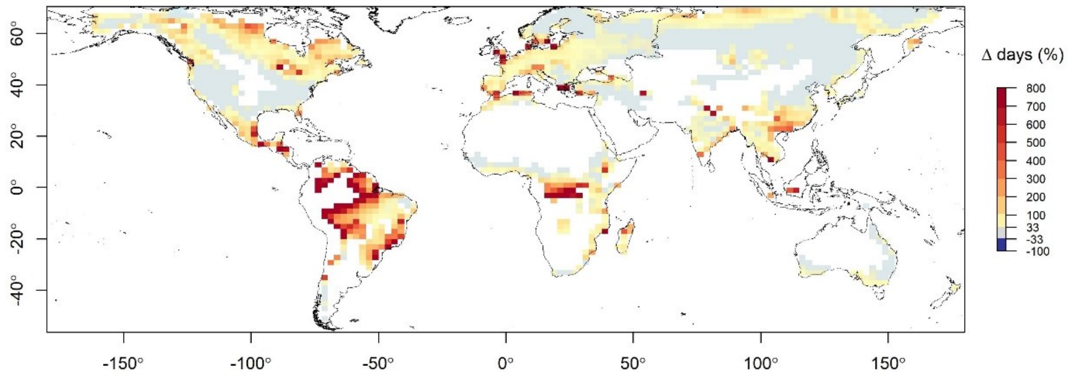

b

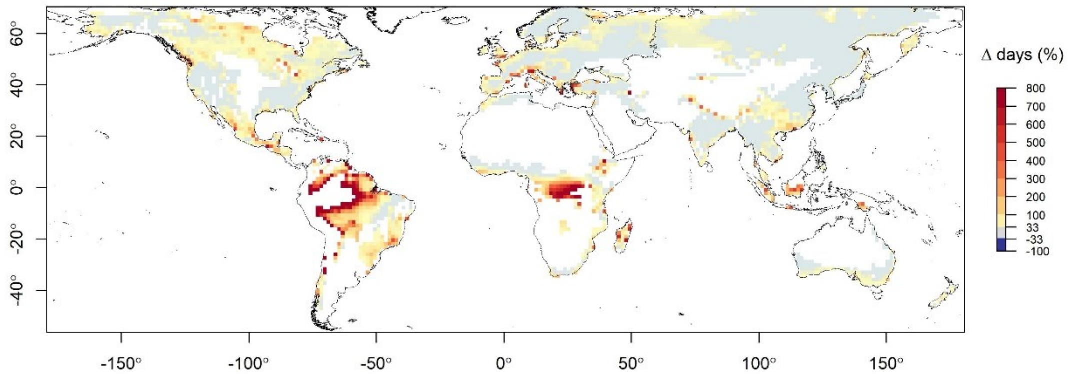

c

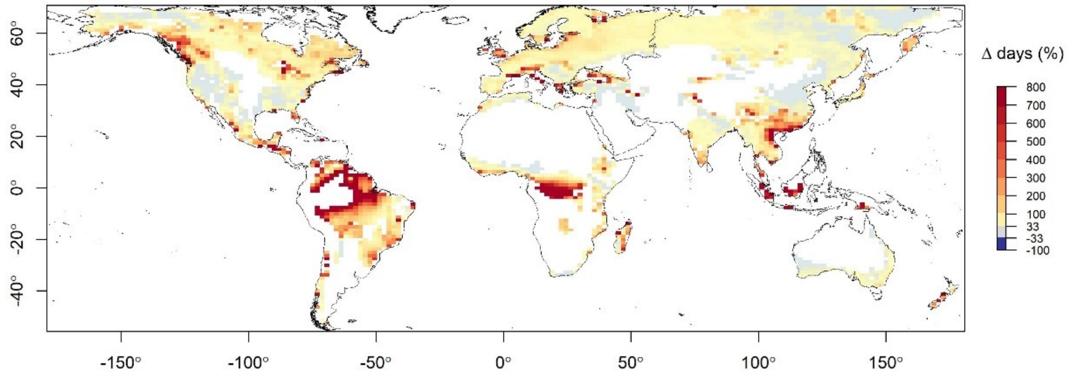

Supplementary Fig. 11 | Projected relative change in the mean annual frequency of days exceeding the VPD thresholds by 2081-2100 under RCP8.5 for the GFDL-CM3 (a), CNRM-CM5 (b) and ACCESS1.0 (c) models. To resolve the majority of the distribution of relative change values, outliers are minimised by capping the scale at 800% (the highest value is 4600%) and omitting pixels with fewer than 5 days per year currently exceeding VPD thresholds. The white areas indicate non-forest land.

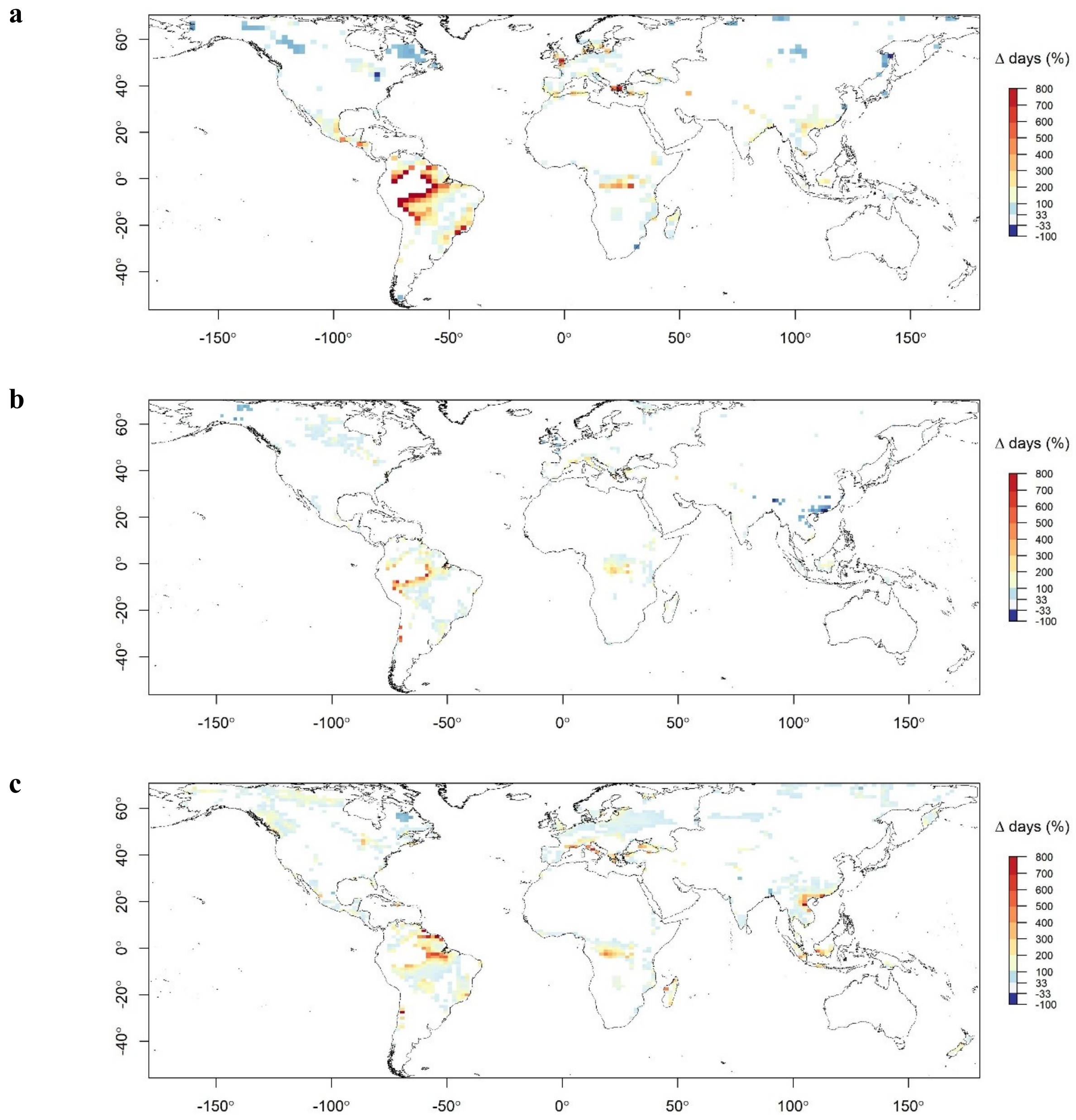

Supplementary Fig. 12 | Projected relative change in the mean annual frequency of days exceeding the VPD thresholds by 2026-2045 under RCP4.5 for the GFDL-CM3 (a), CNRM-CM5 (b) and ACCESS1.0 (c) models. To resolve the majority of the distribution of relative change values, outliers are minimised by capping the scale at 800% (the highest value is 4600%) and omitting pixels with fewer than 5 days per year currently exceeding VPD thresholds. The white areas indicate non-forest land.

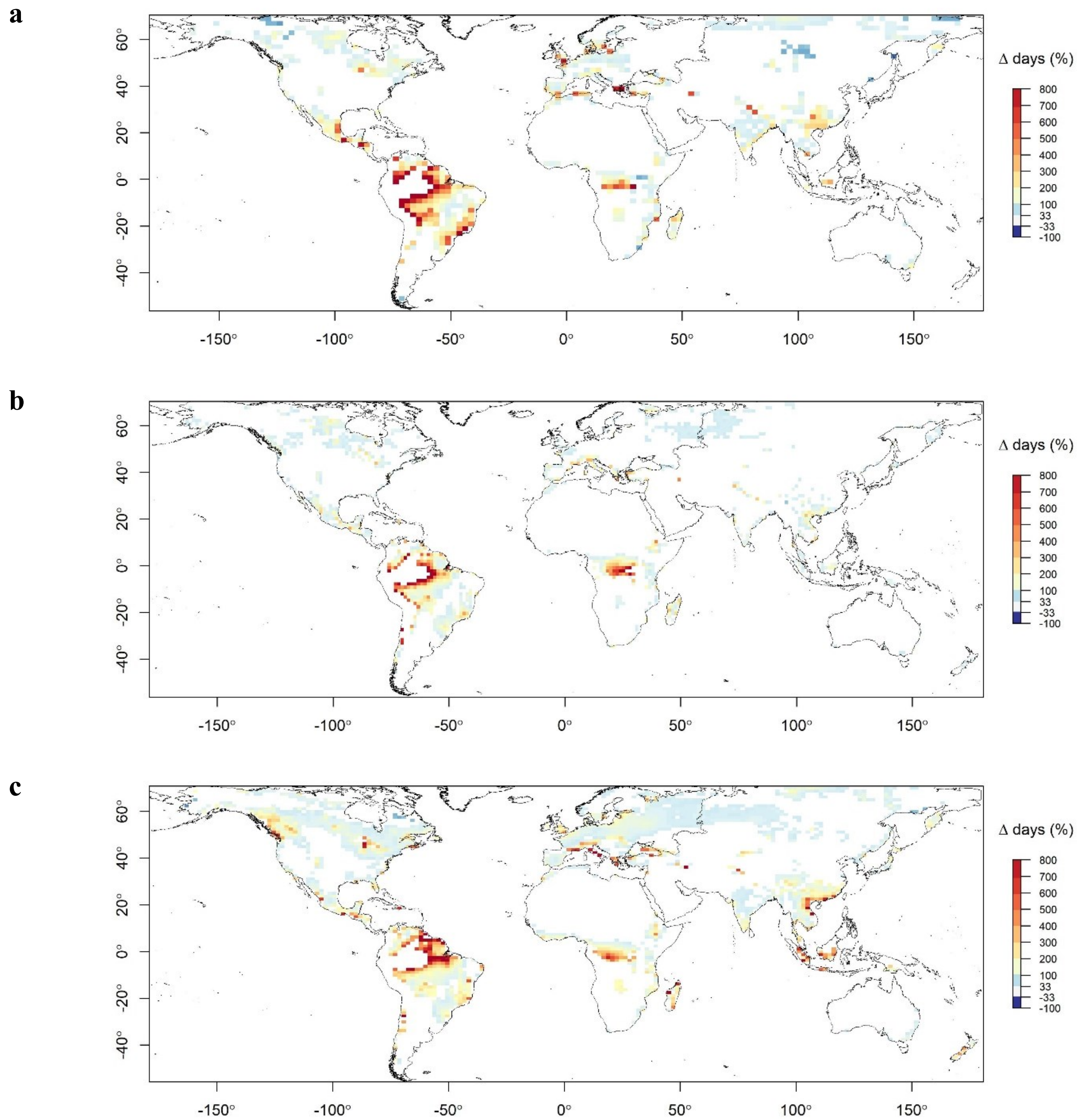

Supplementary Fig. 13 | Projected relative change in the mean annual frequency of days exceeding the VPD thresholds by 2081-2100 under RCP4.5 for the GFDL-CM3 (a), CNRM-CM5 (b) and ACCESS1.0 (c) models. To resolve the majority of the distribution of relative change values, outliers are minimised by capping the scale at 800% (the highest value is 4600%) and omitting pixels with fewer than 5 days per year currently exceeding VPD thresholds. The white areas indicate non-forest land.

References

References

1. Ackerley, D. & Dommenget, D. Atmosphere-only GCM (ACCESS1.0) simulations with prescribed land surface temperatures. *Geoscientific Model Development* **9**(6), 2077-2098 (2016).
2. Fick, S.E. & R.J. Hijmans. WorldClim 2: new 1km spatial resolution climate surfaces for global land areas. *International Journal of Climatology* **37**(12), 4302-4315 (2017).
3. Griffies, S. M., Winton, M., Donner, L. J., Horowitz, L. W., Downes, S. M., Farneti, R., Gnanadesikan, A., Hurlin, W. J., Lee, H. C., Liang, Z., Palter, J. B., Samuels, B. L., Wittenberg, A. T., Wyman, B. L., Yin, J. J., & Zadeh, N. The GFDL CM3 Coupled Climate Model: Characteristics of the Ocean and Sea Ice Simulations. *Journal of Climate* **24**(13), 3520-3544 (2011).
4. McSweeney, C. F., Jones, R. G., Lee, R. W., & Rowell, D. P. (2015). Selecting CMIP5 GCMs for downscaling over multiple regions. Climate Dynamics, 44(11-12), 3237-3260. doi:10.1007/s00382-014-2418-8
5. Voldoire, A., Sanchez-Gomez, E., Melia, D. S. Y., Decharme, B., Cassou, C., Senesi, S., Valcke, S., Beau, I., Alias, A., Chevallier, M., Deque, M., Deshayes, J., Douville, H., Fernandez, E., Madec, G., Maisonnave, E., Moine, M. P., Planton, S., Saint-Martin, D., Szopa, S., Tyteca, S., Alkama, R., Belamari, S., Braun, A., Coquart, L., & Chauvin, F. The CNRM-CM5.1 global climate model: description and basic evaluation. *Climate Dynamics* **40**(9-10), 2091-2121 (2013).
